# Expanded Program on Immunization in the Americas: 40 years

**DOI:** 10.26633/RPSP.2017.139

**Published:** 2017-12-12

**Authors:** Carissa F. Etienne

**Affiliations:** 1 Director, Pan American Health Organization Regional Office of the World Health Organization for the Americas Washington, DC. United States of America Director, Pan American Health Organization, Regional Office of the World Health Organization for the Americas, Washington, DC, United States of America.

Childhood immunization programs have had a dramatic impact on child morbidity and mortality worldwide. In Latin America and the Caribbean, nearly 174 000 deaths were prevented through vaccination of children under 5 years of age in 2006 – 2011 in Latin America and the Caribbean according to Pan American Health Organization (PAHO) estimates (1).

In 1974, the World Health Organization (WHO) established the Expanded Program on Immunization (EPI); and in 1977, PAHO, the WHO Regional Office for the Americas, launched its own EPI. In 1978, a Resolution was approved by the Pan American Sanitary Conference, establishing the working capital for the PAHO Revolving Fund operations (2). The PAHO Revolving Fund—a mechanism facilitating pooled vaccine procurement—was created based on principles of Pan-American solidarity, equitable access to high quality vaccines, and transparency (2). Under the stellar leadership of Dr. Ciro A. de Quadros, the PAHO EPI became the flagship of the Organization (3).

The implementation of the EPI in the Americas marked the birth of a major success story. Immunization coverage increased from 50 % in the 1970s to over 80 % by 1992 (4). In 1994, the Region of the Americas became the first of the WHO regions to eliminate poliomyelitis. Likewise, in 2015 and 2016, the Region of the Americas was declared free of measles, rubella, and congenital rubella syndrome (5), and in 2017, was declared free of neonatal tetanus (6). The introduction of new vaccines (against pneumococcus, rotavirus, influenza, and human papilloma virus, among others) into the EPI’s routine vaccination schedules has been accelerated— using evidence as the basis for decision-making. Meanwhile, the PAHO Technical Advisory Group on Vaccine-preventable Diseases has provided expert oversight of immunization initiatives and policies throughout the Region, setting standard goals and targets for improving immunization coverage, while reviewing and monitoring progress. Furthermore, Vaccination Week in the Americas, which led to the establishment of World Immunization Week, has been a key platform for keeping immunization on the public health agenda and for reaching vulnerable populations.

The progress made in the Americas has been possible through the political commitment and allocated financial resources of PAHO Member States, the exemplary dedication of health care workers, a culture of vaccination created over the years, and the trust that populations have put in their national EPIs. In order to protect this progress and further the impact of EPIs, certain challenges that remain must be addressed: ensuring universal access to vaccines, especially for the most disadvantaged; responding to vaccine hesitancy; maintaining immunization as a high political priority; and ensuring equitable access by managing the high cost of new vaccines.

In 2015, the PAHO Secretariat and Member States committed to fulfilling the mission of the Decade of Vaccines: “to extend, by 2020 and beyond, the full benefit of immunization to all people, regardless of where they are born, who they are or where they live” (7). This commitment was accompanied by a new plan of action for 2016 – 2020 (8) that would guide countries in designing and implementing immunization policies along four strategic lines of action: (i) protect and sustain the achievements, (ii) complete the unfinished agenda, (iii) tackle new challenges, and (iv) strengthen health services for the effective delivery of immunization. The plan, which is being implemented across the Region, urges PAHO Member States to continue to promote a culture of prevention and reduce inequalities by prioritizing the most disadvantaged groups for vaccination, strengthening the public health services infrastructure, continuing to advocate for and consolidating political commitment, and fostering greater integration and universal access to health services. The PAHO Secretariat is working with countries within this framework to strengthen immunization systems as part of the integrated health services, extending services to people currently not covered and using integrated approaches with other interventions at the primary care level.

Immunization programs promote the health and wealth of nations. Vaccination contributes to improved population health, which may potentially translate into lasting, positive impacts on the economy. Ozawa and colleagues, based on the costs of illnesses averted, estimated that immunizations will yield a net return about 16 times greater than the costs of illness (economic burden of avertable deaths, cases, and disabilities) over the decade (uncertainty range: 10 – 25). Bloom and colleagues (10) reported that the impact of vaccination is not solely limited to averting medical costs and illness, but also to improved cognitive development, educational attainment, labor productivity, and income, savings, and investment.

This supplement of the *Pan American Journal of Public Health* highlights key achievements of immunization programs in the Region and their approaches to addressing the current and future challenges, within the framework of the Sustainable Development Goals. This publication offers an opportunity to share the lessons learned and the valuable experiences of one of the world’s leading regions on vaccination.
